# Childbirth preparation and its facilitating and inhibiting factors from the perspectives of pregnant and postpartum women in Tabriz-Iran: a qualitative study

**DOI:** 10.1186/s12978-024-01844-8

**Published:** 2024-07-12

**Authors:** Zohreh Alizadeh-Dibazari, Fatemeh Abbasalizadeh, Sakineh Mohammad-Alizadeh-Charandabi, Shayesteh Jahanfar, Mojgan Mirghafourvand

**Affiliations:** 1 Midwifery Department, Faculty of Nursing and Midwifery, Tabriz University of Medical Sciences, Tabriz, Iran; 2 Women Reproductive Health Research Center, Tabriz University of Medical Sciences, Tabriz, Iran; 3 Midwifery Department, Faculty of Nursing and Midwifery, Tabriz University of Medical Sciences, Tabriz, Iran; 4 Department of Public Health and Community Medicine, Tufts University School of Medicine, Boston, Massachusetts USA; 5 Social Determinants of Health Research Center, Tabriz University of Medical Sciences, Tabriz, Iran

## Abstract

**Background:**

The World Health Organization recognizes childbirth preparation as an essential component of antenatal care, as it plays a crucial role in reducing maternal mortality and improving women's childbirth experience. Countries worldwide have implemented various interventions to assist women in preparing for childbirth, based on their own resources. This study was conducted with the aim of exploring the perspectives of pregnant and postpartum women on childbirth preparation and the facilitating and inhibiting factors, in Tabriz, Iran.

**Methods:**

This qualitative study was conducted with 25 participants, selected purposively among pregnant women in weeks 37 to 40 of gestation and postpartum women within 10 days to 6 weeks after childbirth. Data collection was done through semi-structured, in-depth individual interviews using an interview guide. The data was analyzed using content analysis method with conventional approach.

**Results:**

The perspectives of pregnant and postpartum women regarding childbirth preparation revealed that factors such as maternal health during pregnancy, having an antenatal care plan, improving health literacy, and developing a birth plan were identified as crucial elements for effective childbirth preparation. Additionally, mental and emotional preparation, support, financial planning, participation in preparation classes, and awareness of childbirth methods were recognized as facilitators. On the other hand, insufficient mental and emotional preparedness, inadequate support, weak antenatal care, information deficiencies, insufficient physical activity, and a lack of a birth plan were identified as barriers.

**Conclusion:**

The findings highlight the multifaceted nature of childbirth preparedness, necessitating the involvement of families, the healthcare system, and the entire community. Utilizing the study results in strategic planning for pre-pregnancy, during pregnancy, and inter-pregnancy care can enhance childbirth preparedness and contribute to achieving Iran's population rejuvenation policy goals.

**Supplementary Information:**

The online version contains supplementary material available at 10.1186/s12978-024-01844-8.

## Abstract (highlights)

The World Health Organization recognizes that preparing for childbirth is an important part of prenatal care. It can help reduce maternal deaths and improve women's experiences during childbirth. Countries around the world have implemented different programs to help women prepare for childbirth, based on their own resources. This study was conducted in Tabriz, Iran to understand how pregnant and recently pregnant women view childbirth preparation and what factors help or hinder it. The study involved 25 participants who were intentionally chosen from pregnant women in their last month of pregnancy and women who had given birth within the past 6 weeks by researchers. The researchers conducted in-depth interviews with each participant, using a set of questions, to gather information. From these interviews, the researchers identified several themes related to childbirth preparation. These themes included considering the mother's health during pregnancy, planning for antenatal care, promoting health literacy, creating a childbirth plan, identifying factors that help with preparation, and understanding barriers to preparation. The findings of this study highlight the complexity of preparing for childbirth and emphasize the need for involvement from families, the healthcare system, and the entire community. By utilizing these findings to develop plans prior to pregnancy, during pregnancy, and between pregnancies, we can enhance childbirth preparedness and contribute to Iran's objectives for population growth.

**Supplementary Information:**

The online version contains supplementary material available at 10.1186/s12978-024-01844-8.

## Background

Childbirth is a remarkable yet challenging process of transitioning from a pregnant woman to a mother, creating lifelong memories [1] and impacting a woman's physical and mental health, as well as her bond with her newborn [2]. A positive childbirth experience enhances women's well-being, increases their self-confidence, and facilitates the mother-child relationship. Conversely, a negative childbirth experience can induce psychological distress, lead to postpartum depression, impair the mother-child bond, and hinder the child's normal physical, mental, and emotional development [2, 3]. Every mother desires a childbirth experience filled with beautiful and memorable moments. Achieving this goal requires meticulous planning by and for women to enhance their childbirth preparedness [4]. Indeed, by influencing knowledge, psychological aspects, and birth planning, childbirth preparedness can be augmented [5]. Consequently, women who are prepared for childbirth exhibit improved birthing confidence due to acquired knowledge and can effectively cope with the challenges of pregnancy and labor [6].

Previous studies have established a strong link between adequate childbirth preparedness and reduced maternal complications, diminished fear of childbirth (FoC), and overall positive childbirth experiences [7, 8]. Primiparous women, in particular, require timely access to relevant, appropriate, and accurate information due to their lack of prior experience [9]. Familiarity with the childbirth process and what to expect before and after childbirth can minimize childbirth trauma [4]. Women with less FoC can maintain a positive mindset and focus on childbirth preparedness, leading to enhanced confidence in their ability to handle future pregnancies and deliveries [6]. Conversely, inadequate knowledge and fear of the unknown events associated with pregnancy and childbirth can lead to maternal anxiety and distress [1], negative childbirth experiences, an increased incidence of childbirth trauma, postpartum depression, and post-traumatic stress disorder (PTSD) [10]. FoC is often due to negative experiences or outcomes from previous pregnancies, while for primiparous women, fear of the unknown, pain, injury, loss of the baby, and death are common underlying factors. Considering these factors and others, pregnant women with severe FoC (tokophobia) often prefer cesarean delivery (CD) [11]. However, encouraging and educating mothers prior to childbirth can significantly improve their self-efficacy and preparedness for labor [12].

The World Health Organization (WHO) recognizes childbirth preparedness as a critical component of antenatal care [13], playing a crucial role in reducing maternal mortality and enhancing women's childbirth experiences [14]. Countries worldwide have implemented active interventions to support women in preparing for childbirth. For instance, since 2005, birth preparedness and complication readiness (BP/CR) has been integrated into the antenatal care package in resource-limited countries [15]. BP/CR encompasses a process of planning for childbirth and preparing to take emergency action if necessary, which is crucial for the safety and well-being of mothers and newborns during pregnancy, childbirth, and postpartum period [16]. Developed countries have also established antenatal preparation courses focusing on women's knowledge about childbirth, self-care skills, and self-efficacy, aiming to improve their childbirth experiences and satisfaction [17].

Sustainable Development Goal 3 (SDG3), which aims to end preventable maternal mortality, emphasizes the importance of providing quality care beyond mere access to care [18]. Therefore, conducting childbirth preparedness courses is considered a key objective by the WHO due to the improvement in the quality of antenatal care [19]. However, studies on antenatal care in developing countries reveal that less than 50% of pregnant women receive adequate counseling for childbirth preparation [20], and structured antenatal classes are often not included in the standard package of available antenatal care services [21].

According to the WHO report, Iran is among the nine countries that have achieved the Millennium Development Goal 5A (improving maternal health) with an annual reduction in maternal mortality rate of 6.4% [22]. However, in this context, there has been a sixfold increase in the CD rate from less than 7% in the 1970s to over 48% in 2018, which is due to an increase in elective CDs that are performed without cause and at the request of patients [23]. The Ministry of Health and Medical Education of Iran has been holding childbirth preparation classes across the country since 2008 in order to increase childbirth preparedness and reduce the CD rate [21]. However, despite the holding of these classes and the implementation of the health transformation plan to encourage mothers to have natural childbirth, Iran has not yet reached the target of reducing the CD rate by 10% annually [24]. Based on conducted searches, a qualitative study addressing the perspectives of mothers on childbirth preparedness in Iran was not found. Therefore, this qualitative study was conducted to explore the perspectives of pregnant and postpartum mothers regarding childbirth preparedness and the facilitating and inhibiting factors through interviews using open-ended questions.

## Methods

### Study design

This study is the qualitative part of a mixed-methods study with a sequential explanatory design, and includes two quantitative and qualitative phases. The quantitative part of the study was a descriptive-analytical longitudinal study that examined the status of childbirth preparation and its associated factors and outcomes in pregnant women attending 61 health centers in Tabriz, Iran. The methodology of the qualitative part of this study is content analysis, which was conducted on pregnant and postpartum women from March to September 2023. This qualitative research has been reported based on consolidated criteria for reporting qualitative research checklist (COREQ) [25] (Appendix 1).

### Participants

The participants in this study were purposively selected from pregnant women in weeks 37 to 40 of gestation and postpartum women within 10 days to 6 weeks after childbirth, without any history indicative of high-risk pregnancy, CD, mental illness, or recent adverse events, who fell into the extreme cases category based on their overall childbirth readiness score (CRS) calculated in the quantitative section (scores at both ends of the score spectrum based on the CRS questionnaire), and were willing and able to describe their experiences regarding childbirth preparedness. The women's willingness to participate in the study was assessed through telephone contact with the researcher. If they were willing to participate, a face-to-face meeting at their preferred location was arranged, during which the study's objectives and implementation process, including their rights and confidentiality of interview content and recordings, were explained, and informed written consent was obtained from the participants. None of the participants refused or left the study.

### Data collection

Data were collected through individual in-depth semi-structured interviews using open-ended questions. The interviews were conducted by the first author (Z A-D), who is a PhD student in midwifery and has 20 years of teaching experience in the field of providing care to mothers during pregnancy, childbirth, and postpartum. The interview began with the main question "What actions have you taken to prepare for childbirth?" and then continued with other questions depending on the participants' responses, such as "What factors do you think contribute to enhancing childbirth preparedness?" and "What factors do you think hinder childbirth preparedness?" In addition, based on the type of response to each question, probing and exploratory questions were asked to gain a deeper understanding of the women's experiences, such as "What do you mean?" "Why?" "Can you explain more?" "Can you provide an example to help me understand?" Non-verbal data such as tone of voice, facial expressions, and participants' body language were also recorded by the researcher during the interview on a specific sheet, noting the time and place of the interview. The interview location was chosen at the mother's discretion and was generally conducted at the health center where the mother had an electronic file. The duration of the interviews ranged from 30 to 60 minutes, and with the participants' consent, the interviews were audio-recorded.

### Data analysis

Qualitative content analysis using the conventional approach, as proposed by Graneheim and Lundman [26], was employed to analyze the data. With this method, in addition to the explicit content of the interview texts, the hidden content and concepts with different levels of abstraction can also be achieved. Therefore, based on this method, five steps were taken: Transcribing the entire interview immediately after each interview; Reading the entire text several times to gain a general understanding of its content; Dividing the text into meaning units, extracting a summary of the meaning units, and coding; Classifying the initial codes into sub-themes based on comparing their similarities and differences; and Extracting themes as expressions of the latent content and concepts in the data. In this study, after transcribing the interview texts, 95 pages of raw data were obtained. The data analysis was conducted by two individuals, including the first author (Z A-D) and the corresponding author (M M). Phrases and sentences related to the actions taken by mothers to prepare for childbirth were coded in the margins of the transcribed pages. The coding was primarily close to the text and reflected the mothers’ own explanations. Subsequently, codes with similar content were combined to form sub-themes and themes. The authors discussed and exchanged views on the interpretations of the actions taken by mothers to prepare for childbirth and reached a consensus on the themes.

### Trustworthiness of the findings

To increase the trustworthiness of the data, the five criteria of dependability, credibility, transferability, confirmability, and authenticity were considered in the research [27]. To ensure the dependability of the data, maximum diversity in terms of age, education, employment status, and socioeconomic status was considered in the selection of participants. To facilitate the collection of credible data, conditions were created to allow participants to express their opinions freely and openly by creating a sense of trust and comfort during the interview. In addition, three participants were interviewed in two stages before and after childbirth and their interview transcripts were compared. Also, in this research, Member Checks were used in such a way that after transcribing the interviews and coding the interview texts, the coded text was given to 3 participants and they were asked to confirm the accuracy of the content and the relevance of the extracted codes with their experiences and opinions. To increase transferability, it was possible to judge the extent to which the research context fits with other contexts. This was done by including detailed descriptive information, i.e., a rich and extensive set of details about the method and context, in the research report. To increase confirmability, all research steps were clearly documented so that other researchers could also follow up on the data. To increase the authenticity of the data, at least one relevant quote was provided for each identified subtheme. In addition, quotes from different participants were also included.

## Results

After interviewing 22 participants, data did not introduce new codes but rather reinforced what had already been observed. This indicated that the data had become saturated, so the interviews were completed with the 25^th^ participant. The participants were selected from 15 health centers located in Tabriz, Iran, and were between 18 and 39 years of age and had education levels ranging from illiterate to university. Participants included 10 pregnant women and 15 postpartum women, and their pregnancy history ranged from one to four pregnancies (Table 1).

**Table 1:** Sociodemographic characteristics of participants

**Participants**^**†**^	**Age** (year)	**Education**	**Occupation**	**Number of pregnancies**	**Number of parities**	**Time of interview**
P1	20	Diploma	Housewife	1	0	Pregnancy
P2	30	University	Housewife	1	0	Pregnancy
P3	28	Secondary school	Housewife	1	0	Pregnancy
P4	19	High school	Housewife	1	1	Postpartum
P5	32	University	Teacher	2	1	Postpartum
P6	25	Secondary school	Housewife	2	1	Pregnancy
P7	29	Secondary school	Housewife	3	1	Pregnancy
P8	20	High school	Housewife	2	1	Postpartum
P9	27	University	Housewife	1	0	Pregnancy
P10	20	Diploma	Housewife	1	0	Pregnancy
P11	28	Secondary school	Housewife	2	2	Postpartum
P12	19	Secondary school	Housewife	1	1	Postpartum
P13	28	Primary school	Housewife	3	3	Postpartum
P14	39	Illiterate	carpet weaver	3	3	Postpartum
P15	37	Diploma	Housewife	2	1	Postpartum
P16	26	Diploma	Housewife	1	0	Pregnancy
P17	23	Diploma	Housewife	2	1	Pregnancy
P18	24	Diploma	Housewife	2	2	Postpartum
P19	18	High school	Student	1	1	Postpartum
P20	34	Secondary school	Housewife	3	3	Postpartum
P21	19	High school	carpet weaver	1	1	Postpartum
P22	25	High school	Housewife	3	2	Postpartum
P23	33	High school	Housewife	2	2	Postpartum
P24	23	Diploma	Housewife	1	1	Postpartum
P25	29	High school	Housewife	5	4	Pregnancy

**Fig. 1:**
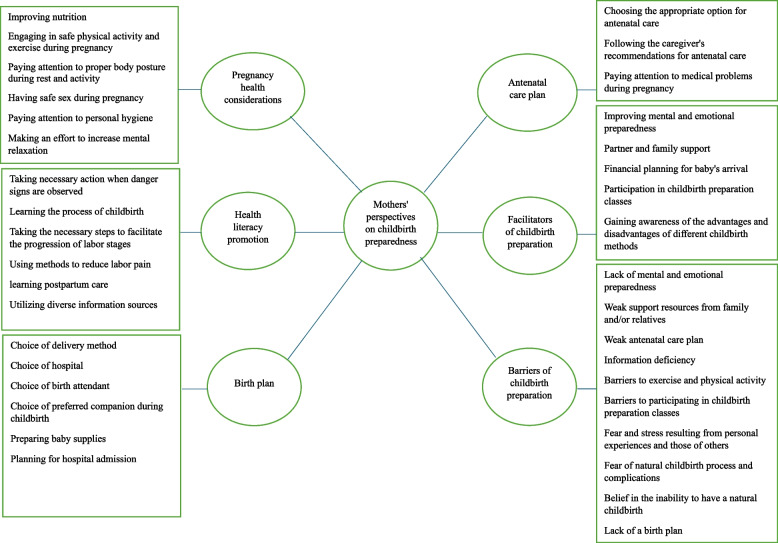
Classification of main themes and sub-themes

Through the analysis of mothers' perspectives on childbirth preparedness, 290 codes were extracted, which were divided into 36 sub-themes and 6 main themes. An example of the content analysis process, including coding, subthemes, and main themes, is presented in Table 2. The six main themes extracted were "Pregnancy health considerations", "Antenatal care plan", "Health literacy promotion", "Birth plan", "Facilitators of childbirth preparation", and "Barriers to childbirth preparation" (Figure1). For clarity, the main themes and sub-themes were presented separately, and direct quotes were used to demonstrate how participants' perspectives supported these themes. To maintain confidentiality, numerical identifiers (e.g., Participant 1) were used instead of participants' names in the quoted text.

**Table 2:** An example of the analysis process

**Meaning unit**	**Code**	**Sub-theme**	**Theme**
In anticipation of a natural childbirth, I made an effort to increase my walking routine to facilitate a smoother delivery.	Engaging in regular walks driven by a desire for a natural childbirth experience	Engaging in safe physical activity and exercise during pregnancy	Pregnancy health considerations
During my walks, I always carried water and ensured adequate hydration by consuming plenty of fluids.	Carrying and consuming water during walks		
Adhering to my doctor's advice, I ensured to avoid overexertion during my walks.	Avoiding strenuous walks that lead to exhaustion

## Pregnancy health considerations

In the present study, among the necessary measures to improve childbirth preparedness from the perspective of pregnant and postpartum women, the importance of paying attention to their health during pregnancy has been mentioned from various angles. The sub-themes include "Improving nutrition", "Engaging in safe physical activity and exercise during pregnancy", "Paying attention to proper body posture during rest and activity", "Having safe sex during pregnancy", "Paying attention to personal hygiene", and "Making an effort to increase mental relaxation".

### Improving nutrition

According to pregnant and postpartum women in this study, consuming nutritious foods, especially milk and other dairy products, fruits, vegetables, animal and plant proteins, and plenty of fluids, avoiding salty, fatty, and preservative-laden foods, and limiting the intake of sugary foods particularly for mothers whose blood sugar tests during pregnancy were above the normal range, were mentioned as ways to improve childbirth preparedness.

"I used to drink three glasses of milk a day, or a bowl of yogurt instead. I would eat 100 grams of red meat or chicken, and on days when we didn't have meat, I would eat beans instead. I would consume a bowl of lentils per week and alongside lunch and dinner, I would use vegetables. I consumed a lot of fruits and drank plenty of fluids. I avoided excessive consumption of sweets, salty, and fatty foods." (P4)

### Engaging in safe physical activity and exercise during pregnancy

From the perspective of pregnant and postpartum women, avoiding excessive rest and engaging in physical activities such as housework, walking (especially in the later months of pregnancy), and pregnancy exercises with safety precautions (adequate fluid intake, preventing fatigue, avoiding physical activity in hot weather, avoiding strenuous exercise, and walking if the baby has slow growth) were considered effective in improving childbirth preparedness. Additionally, perineal massage, performing pelvic tilts, and stepping up and down a low platform as actions that lead to improved preparedness for childbirth and facilitate the natural childbirth process were mentioned.

"Because I wanted to have a natural childbirth, I tried to walk more to make my labor easier. I always carried water with me during walks and drank plenty of fluids. Following the doctor's advice, I tried not to tire myself during walking, and whenever I felt tired, I would rest." (P22)

### Paying attention to proper body posture during rest and activity

Mothers emphasized the importance of maintaining good posture during rest, such as sleeping on the side, especially the left side, and avoiding sleeping on the back. They also stressed the importance of good posture during activity, such as sitting, standing up, and going up and down stairs, during pregnancy to improve childbirth preparation.

"At the health center, they had instructed me not to sleep on my back because it would prevent enough blood from reaching the baby. They also said I shouldn't sleep on my stomach and that it was better to sleep on my side, especially my left side. So, I didn't sleep on my back and tried to sleep on my side." (P21)

### Having safe sex during pregnancy

Regarding sexual intercourse during pregnancy, the mothers emphasized the importance of having intercourse based on their own desire and with safety precautions, such as avoiding frequent intercourse, especially in late pregnancy.

"My husband asked me to ask the midwife at the health center about having sex, and she said it depends on my tolerance, but too much sex is harmful. She also said that it is better to avoid sex in late pregnancy. We followed her advice." (P21)

### Paying attention to personal hygiene

To improve childbirth preparation, the mothers mentioned the importance of personal hygiene during pregnancy, including daily bathing, frequent changes of underwear, careful use of detergents, and genital hygiene to prevent genital infections. They also emphasized the importance of oral hygiene during pregnancy by brushing regularly, flossing, and visiting the dentist while informing them of their pregnancy.

"I pay a lot of attention to my hygiene. I take showers frequently. I change my underwear several times a day. I keep my genital area dry. I had burning and itching early in my pregnancy, and my doctor recommended keeping my genital area dry. Following their advice, I have felt much better." (P1)

"I was careful not to let my teeth decay. I brushed regularly, but I couldn't use dental floss because my gums were hurting. Despite brushing, unfortunately, two of my teeth decayed. Following my doctor's advice, I went to the dentist." (P19)

### Making an effort to increase mental relaxation

Mothers emphasized efforts to increase mental tranquility during pregnancy to improve childbirth preparation, through continuing normal life, having a regular sleep schedule, removing sources of stress from their lives, focusing on the positive aspects of pregnancy, and not focusing on the negative aspects such as pregnancy complications and childbirth difficulties.

"I tried not to think about childbirth at all from the beginning of pregnancy because thinking about it caused stress for me. I wanted my pregnancy to progress and see what happens in the end. I just wanted to be relaxed and keep negative thoughts away from me." (P5)

## Antenatal care plan

In this study, having an antenatal care plan was mentioned as one of the ways to improve childbirth preparedness from the mothers' perspective. This included the subthemes of "Choosing the appropriate option for antenatal care", "Following the caregiver's recommendations for antenatal care", and "Paying attention to medical problems during pregnancy".

### Choosing the appropriate option for antenatal care

Prenatal care is provided in public centers, including health centers, clinics, and specialized and super-specialized hospitals, as well as in private centers. Choosing the right option for prenatal care is considered effective by most mothers for improving childbirth preparation. This is based on different criteria, including the distance from home to the center, the cost of services, satisfaction with the center in previous visits, recommendations from friends and family, and recommendations from healthcare providers.

"The health center was not close to my house. I went to a doctor's office near my house for prenatal cares. I had pain at week 31 and my doctor said there was a chance of premature labor and sent me to a super-specialized hospital for a check-up. Since then, I have been going to the same hospital for prenatal cares." (P10)

### Following the caregiver's recommendations for antenatal care

Mothers emphasized the importance of acting on caregiver recommendations, such as regular prenatal cares, regular intake of supplements, undergoing all tests, ultrasounds, screenings, and receiving necessary vaccinations during pregnancy, including tetanus and influenza vaccines, to improve childbirth preparation.

"I regularly went for prenatal check-ups. In early pregnancy, I went once a month, then every two weeks, and in late pregnancy, I went every week for check-ups. I took the prescribed supplements. I underwent all the tests and ultrasounds recommended by my caregiver." (P22)

### Paying attention to medical problems during pregnancy

Mothers emphasized the importance of paying attention to medical problems during pregnancy, including thyroid disorders, blood sugar disorders, and blood pressure disorders, and seeing a doctor to manage these disorders in order to prepare for childbirth.

"In the middle of my pregnancy, I found out that I had a thyroid disorder during the tests that my doctor ordered. My doctor sent me to an internist and they started me on medication and emphasized that I take it every day." (P3)

## Health literacy promotion

According to pregnant and postpartum women in this study, improving health literacy was identified as one of the ways to improve childbirth preparation. The subthemes of this include: "Taking necessary action when danger signs are observed", "Learning the process of childbirth", "Taking the necessary steps to facilitate the progression of labor stages", “Using methods to reduce labor pain”, "Learning postpartum care", and “Utilizing diverse information sources”.

### Taking necessary action when danger signs are observed

From the perspective of pregnant and postpartum mothers, being aware of the essential actions required in case of danger signs during pregnancy is essential for every pregnant woman and improves preparation for childbirth. Some of the issues mentioned by the mothers in this study include how to detect a decrease in fetal movement, measuring blood pressure when feeling a headache, blurred vision, and epigastric pain, and going to the hospital if there is a decrease in fetal movement, abdominal pain, bleeding, leakage, headache, blurred vision, epigastric pain, painful urination, and frequent urination.

"I had learned from childbirth preparedness classes that whenever I felt the baby's movements were reduced, I would eat a sweet food like cake or dates, lie on my left side, and count the baby's movements for an hour. If I felt the baby's movements less than four times in an hour, I would go to the hospital." (P11)

### Learning the process of childbirth

In this study, mothers considered familiarity with the process of childbirth and its stages as effective in preparing for childbirth. Through participating in childbirth preparedness classes, watching natural childbirth videos on the internet, previous experience of natural childbirth, and studying the stages of childbirth from various sources, mothers were acquainted with the stages of childbirth.

"By watching childbirth videos on the internet, I learned that the cervix is closed before the onset of labor and gradually opens as labor pains begin. In the early stages of labor, there is mild back pain and abdominal pain that comes and goes. Gradually, the pains increase until the cervix is fully dilated to 10 centimeters. At this stage, the baby is delivered by pushing." (P8)

### Taking the necessary steps to facilitate the progression of labor stages

The mothers expressed that their familiarity with the actions they should take upon observing labor signs, before going to the hospital, and during hospitalization in the first and second stages of labor was effective in improving their preparedness for childbirth. They emphasized that distinguishing true labor pains from false ones, taking a shower, exercising, walking, and going to the hospital with intensifying labor pains and decreasing intervals between contractions leads to a reduction in the duration of hospitalization. Mothers mentioned that taking a shower, walking in the delivery room, especially with long strides, and performing specific exercises such as sitting on a Pilates ball and doing pelvic and lumbar rotational movements during the first stage of labor were beneficial. They also noted that restlessness and shouting during childbirth lead to the wastage of body energy and feelings of fatigue. Mothers considered following the midwives’ recommendations in the delivery room to be effective in improving the progress of labor. Mothers emphasized the importance of timing and proper method of pushing, breathing technique during pushing, the direction of pushing, and the body position during pushing.

"When I was admitted, they told me that my labor might take a long time because my cervix was not dilated. But the staff in the delivery room taught me to walk, exercise, and take hot showers in the delivery room. I tried to follow their advice. Within five hours, my cervix was dilated." (P5)

### Using methods to reduce labor pain

From the mothers' point of view, although pain is an inseparable part of the labor process, being aware of some non-pharmacological measures that lead to a reduction in labor pain, such as back and lower back massage, acupressure, smelling lavender essence, and focusing on the type of breathing, can be effective in improving preparation for childbirth. Mothers also mentioned that being assured of the availability of pharmacological methods of labor pain relief in the hospital makes it easier to choose natural childbirth and improves preparation for childbirth.

"The midwife who was taking care of me in the delivery room taught me a type of breathing to do during pain. By doing this type of breathing, my pain was reduced and my cervix opened very easily and I gave birth." (P21)

### Learning postpartum care

Newborn care education, such as breastfeeding, umbilical cord care, infant sleep, bathing techniques, and recognizing illness symptoms like jaundice, along with postpartum self-care education, including proper nutrition, maintaining personal hygiene, caring for stitches, breast care, postpartum exercises, and the need for increased spousal support during this time, particularly for first-time mothers, were identified as factors influencing mothers’ childbirth preparation.

"I cared for my sister after her delivery last year. That's when I discovered that the baby's umbilical cord shouldn't be placed in the diaper and should be left exposed to air. I also learned that burping the baby after feeding helps prevent stomach discomfort." (P20)

"Childbirth preparation classes taught us to avoid cold air exposure after delivery and to clean our stitches with saline solution. They stressed that excessive visitors during this time are not recommended due to the increased risk of infectious diseases for both mothers and babies, and the greater presence of our spouse by our side is beneficial." (P2)

### Utilizing diverse information sources

The presented study explores the diverse information sources utilized by pregnant and postpartum mothers to enhance their health literacy and prepare for childbirth such as personal and others experiences, social networks, healthcare provider educations, and written resources such as books and the internet.

"I have previously had a natural childbirth and have the experience of natural childbirth. It is true that my previous childbirth was difficult, but I was very comfortable after giving birth. That's why I am familiar with the stages of childbirth and I know that I have to push during the pain and breathe in between contractions." (P6)

"I obtained my information about pregnancy and childbirth stages from an application called 'Gahvareh' that I installed on my mobile phone. In this application, pregnant and postpartum women communicate with each other and share their experiences. I learned through this app that I should exercise, climb stairs, and walk to increase labor pains and expedite delivery. I did these things, and they were very effective." (P8)

## Birth plan

In the present study, through examining the perspective of pregnant and postpartum mothers, having a birth plan emerged as another strategy for improving childbirth preparedness. Subthemes included "Choice of delivery method", "Choice of hospital", "Choice of birth attendant", "Choice of preferred companion during childbirth", "Preparing baby supplies", and "Planning for hospital admission".

### Choice of delivery method

Among the various decisions expectant mothers make in preparation for childbirth, choosing the mode of delivery appears to be the most challenging. Exploring the perspectives of mothers revealed that several factors significantly influence their decision-making process. These factors include: weighing the pros and cons of each delivery method, prior delivery experiences, physician's recommendations, cost considerations, and partner's preferences.

"Since my first child was delivered naturally, I have decided to have this one naturally as well. Cesarean delivery is very expensive, and I don't want to put a financial burden on my husband." (P7)

### Choice of hospital

Choosing a suitable hospital for childbirth presents another challenge for expectant mothers during their preparation journey. Exploring mothers' perspectives revealed several factors such as delivery method compatibility, cost considerations, hospital distance, recommendations from physician and others, and prior hospital experiences influence their hospital selection decisions.

"There are both private and public hospitals near my home. Due to the high cost of private hospitals, I decided to go to a public hospital. During my first visit, I noticed the overcrowding and lack of adequate care at the hospital, so I decided to go to another public hospital that was further away, even though the commute was very difficult." (P15)

### Choice of birth attendant

In planning for childbirth, mothers emphasized the importance of choosing their own birth attendant as a crucial aspect of preparation. However, the lack of this option in public hospitals was identified as a source of confusion and concern.

"Choosing the obstetrician for my baby's health is important to me, and I'd like to make the choice myself, but I know it's not possible in public hospitals. I'm afraid something might go wrong during natural childbirth." (P7)

### Choice of preferred companion during childbirth

The mothers emphasized the importance of the presence of their preferred companion during childbirth, as it contributed to improving their preparedness for childbirth. In their childbirth plans, they often included arrangements for their mother, sister, spouse, friends or acquaintances with experience in natural childbirth, or a doula (accompanying midwife) to be present.

"I felt very alone in the delivery room. The midwife allowed my sister to come in with me. My sister has had two natural childbirths before, and her presence was very helpful for me. It's true that my husband wasn't with me in the delivery room, but his presence outside the room was a source of strength for me." (P5)

### Preparing baby supplies

In childbirth planning, mothers emphasized the importance of purchasing newborn essentials and preparing the hospital bag with necessary items a few weeks before the due date.

"From the time I found out the baby's gender, I slowly started buying baby items, and I had prepared the hospital bag a month before childbirth." (P8)

### Planning for hospital admission

In planning for hospital visits, mothers emphasized the importance of coordinating with their spouses, family, friends, and acquaintances regarding timely hospital visits by observing danger signs and childbirth symptoms, considering the distance to the hospital, and making prior arrangements for the necessary transportation for the mother to the hospital.

"I had instructed my husband that whenever I experience labor symptoms, I need to go to the hospital. And if he's not available, I will go to the hospital with my mother and contact him to come to the hospital." (P12)

## Facilitators of childbirth preparation

An exploration of mothers' perspectives on childbirth preparedness revealed several key factors that facilitated their preparation journey. These subthemes can be summarized as "Improving mental and emotional preparation", "Partner and family support", "Financial planning for baby's arrival", "Participation in childbirth preparation classes", and "Gaining awareness of the advantages and disadvantages of different childbirth methods".

### Improving mental and emotional preparedness

From the perspective of mothers, focusing on the joy of giving birth and motherhood, drawing from positive prior experiences, avoiding stressful sources such as stressful remarks from acquaintances, striving to increase calmness through participation in counseling sessions and reading the Quran, and focusing on encouraging words from the doctor and acquaintances lead to an increase in mental and emotional childbirth preparation.

"Although natural childbirth is very difficult, the sweetness of giving birth to a child outweighs its difficulty. Giving birth to my child enables me to endure this pain." (P2)

### Partner and family support

The mothers emphasized the significant impact of their husbands’ and families’ support, especially older children at home, mothers, sisters, and in-laws, in facilitating childbirth preparation. This support included assisting with household chores, accompanying them to prenatal visits, ultrasounds, and tests, attending childbirth preparation classes, learning about danger signs and labor symptoms, and understanding the need to go to the hospital if these signs are observed. Additionally, they provided advice to mothers in case of problems during pregnancy, strived to maintain their mental peace, offered financial support, helped them make decisions such as choosing the type of delivery, supported their decisions during this period, and reassured mothers about their presence in the delivery room and care for them and their newborns after delivery.

"I have taught my husband the danger signs. He measures my blood pressure at home, asks me about back pain and abdominal pain, and controls the baby's movements by putting his hand on my stomach. My husband is very supportive of me mentally. In addition, they help me a lot with housework and cooking." (P17)

"I don't have a mother. I only have one sister who helps me a lot. She has told me to let her know whenever I have pain. In addition, she has promised to help me take care of the baby." (P7)

### Financial planning for baby's arrival

Mothers emphasized that having financial planning and saving money during pregnancy to cover the costs of prenatal visits, ultrasounds and tests during this period, purchasing necessities for themselves and their newborns, and covering hospital costs at the time of delivery facilitates preparation for childbirth.

"During pregnancy, my husband and I tried to save up to buy a personal car. We thought having a personal car would be necessary both for the time of delivery and for after delivery. We managed to buy a car in late pregnancy." (P16)

### Participation in childbirth preparation classes

From the mothers' perspective, attending childbirth preparation classes leads to improved childbirth preparation by increasing knowledge, reducing fear and stress, and boosting confidence in natural childbirth.

"At first, I was afraid of natural childbirth, but I had heard that the complications of natural childbirth are very low. So, I decided to take natural childbirth classes to learn more. Attending these classes helped me increase my knowledge about childbirth, eliminate my fear, and now I feel very hopeful and relaxed." (P2)

### Gaining awareness of the advantages and disadvantages of different childbirth methods

Mothers emphasized that being aware of the advantages and disadvantages of each type of delivery leads to easier decision-making regarding the mode of delivery and also improves preparation for childbirth. Significantly less postpartum pain and bleeding during delivery in natural childbirth compared to CD, the baby being more alert, intelligent, and healthier in natural childbirth compared to CD, the possibility of better care for the baby after natural childbirth compared to CD, and the different course of natural childbirth in different individuals were among the reasons mentioned by mothers for choosing natural childbirth. They also mentioned that CD is performed in cases where natural childbirth is not possible and in cases where the lives of the mother and baby are at risk.

"I have heard that the pain of natural childbirth lasts for a few hours at most, but in cesarean delivery, despite the absence of pain during delivery, there is severe pain after delivery and it leaves many complications. So I have decided to have a natural childbirth because I prefer not to have pain after delivery when the baby needs to be breastfed and cared for." (P9)

## Barriers to childbirth preparation

The study of mothers' perspectives on childbirth preparation identified several factors that hinder childbirth preparation. These subthemes include: "Lack of mental and emotional preparedness", "Weak support resources from family and/or relatives", "Weak antenatal care plan", "Information deficiency", "Barriers to exercise and physical activity", "Barriers to participating in childbirth preparation classes", "Fear and stress resulting from personal experiences and those of others", "Fear of natural childbirth process and complications", "Belief in the inability to have a natural childbirth", and "Lack of a birth plan".

### Lack of mental and emotional preparedness

From the mothers' perspectives, factors such as unplanned pregnancy, distress of older children in the family due to the mother's pregnancy, having a previous unsuccessful pregnancy experience, considering a CD from the beginning of pregnancy and not thinking about natural childbirth, and having a lot of stress as the time of delivery approaches were identified as factors contributing to the lack of emotional preparedness for childbirth.

"During the mid-pregnancy ultrasound, the baby's head was up, and my doctor said that if this condition persists, I would have to have a cesarean delivery. As a result, I didn't even think about natural childbirth. But the last month's ultrasound showed that the baby's head was down, and my doctor recommended natural childbirth. I was shocked because I hadn't planned for natural childbirth at all and I wasn't emotionally prepared for it." (P16)

### Weak support resources from family and/or relatives

Based on the mothers' perspectives, factors such as lack of support from the husband due to his busy schedule or being away from the family, lack of support from the family due to their death, long distance, and being busy were identified as weaknesses in support resources that hinder childbirth preparation.

"My husband is a drug addict and has no job. He has been in a rehabilitation center for the past month. My mother is also poor, and my brother sometimes helps me financially. My pregnancy was unplanned. I tried to abort the baby until the last month of pregnancy, but it didn't work. During pregnancy, I went to the health center for check-ups occasionally." (P13)

### Weak antenatal care plan

Based on the mothers' perspectives, factors such as long distance to healthcare facilities, inadequate care and education, and the unavailability of necessary supplements at health centers were identified as weaknesses in prenatal care that hinder childbirth preparation.

"I went to the health center early in my pregnancy to open a file, but I didn't go back later because of the long distance and also because they didn't provide any special care other than measuring my weight and blood pressure." (P9)

### Information deficiency

Based on the mothers' perspectives, information deficiencies were identified as barriers to childbirth preparation due to factors such as the inability to use information available on the internet and social media due to low literacy, embarrassment to talk to others about pregnancy and childbirth issues, inability to learn materials due to the large volume of information, and forgetting learned materials in the delivery room due to high stress when facing a new environment and staff. Mothers emphasized the importance of repeated repetition of the necessary learning materials during prenatal visits and re-reminding them in the delivery room.

"When entering the delivery room, due to high stress and unfamiliarity with the environment and hospital staff, I became confused and forgot the material I had learned in the educational classes. I would have liked to be reminded of the things I needed to do at each stage so that I could do them." (P14)

### Barriers to exercise and physical activity

In examining the perspective of mothers, barriers to exercising and physical activity during pregnancy were considered as inhibitors of childbirth preparation. These include feelings of physical incapacity, experiencing pain during physical activity, lack of sufficient time due to excessive busyness, and the belief that physical activity and exercise do not contribute to childbirth preparation.

"I used to walk a lot during my first pregnancy, but I couldn't walk in the later months of this pregnancy because the baby's head was pressing on my pelvis and my vagina was stretching." (P18)

### Barriers to participating in childbirth preparation classes

Based on the mothers' perspectives, factors such as lack of information about the time and location of childbirth preparation classes, the long distance to the class location, the busy schedule of the mother or husband or both, the need for more rest at home as recommended by the doctor, and the decision to have an elective CD were identified as barriers to attending childbirth preparation classes and were considered as hindrances to childbirth preparation.

"The midwife at the health center advised me to attend childbirth preparation classes that were held at another health center. However, I couldn't attend despite my desire because the class location was further away from our home." (P2)

### Fear and stress resulting from personal experiences and those of others

Based on the mothers' perspectives, fear and stress arising from personal experience of a difficult natural childbirth, witnessing the labor stages of women in labor in the same hospital room, hearing stories from others about difficult natural childbirth, and the long time that has passed since the person's previous childbirth were identified as barriers to childbirth preparation.

"In the delivery room, in the bed next to me, a pregnant woman was lying down, who had been hospitalized and in pain for three days but had not given birth yet. Seeing her made me feel scared because I was thinking about how long I would have to endure pain before I could give birth." (P14)

### Fear of natural childbirth process and complications

Mothers’ perspectives revealed that fear of the natural childbirth process and its complications, such as the possibility of sudden and unexpected onset of labor symptoms, labor pain and a perceived inability to withstand it, undergoing repeated vaginal examinations during labor, the possibility of hospital staff applying pressure on the mother’s abdomen during childbirth, the possibility of episiotomy and suturing of the external genitalia, complications for the baby during natural childbirth, and the possibility of having a CD after enduring the pains of natural childbirth due to difficulties in the natural childbirth process. Additionally, concerns about pelvic floor muscle relaxation and its consequences, such as urinary and fecal incontinence, decreased sexual satisfaction for both the mother and spouse, and the need for surgery to address these issues, lead to reduced preparation of women for childbirth.

"The fear of the agony and pain of labor makes me feel less prepared. I don't think I can handle this pain and I don't see in myself the ability to endure the pains of labor." (P1)

"I have heard that after natural childbirth, there can be tearing and widening of the vagina, and the surgery to repair this tearing is quite expensive. Without surgery, the vagina remains widened, allowing air to enter the uterus and causing difficulties in sexual relations with the spouse. Therefore, I think that despite its many complications, cesarean delivery is better than natural childbirth." (P2)

### Belief in the inability to have a natural childbirth

Mothers' beliefs about their inability to undergo natural childbirth due to various reasons, such as inadequate nutrition during pregnancy, insufficient weight gain during pregnancy, absence of labor pains despite reaching the due date, and failure of the cervix to dilate despite reaching the due date, were classified as barriers to childbirth preparation.

"Unlike other pregnant women, I struggled to maintain a healthy diet and gain adequate weight during pregnancy. Consequently, I doubt my ability to endure the physical demands of natural childbirth." (P10)

### Lack of a birth plan

Upon examining mothers' perspectives, the lack of a birth plan such as not knowing the expected due date and being surprised during hospitalization, not choosing a hospital and going to the nearest hospital when labor symptoms start, arriving late at the hospital due to a lack of coordination for accessing transportation, and the late arrival of the accompanying person to the hospital due to lack of prior coordination with them, were classified as inhibitors of childbirth preparation.

"I don't have anyone in this city, and my husband is in a rehab camp. When my labor pains started, my daughter called our neighbor. The neighbor took the car and brought me to the first hospital on the way. I had no knowledge of that hospital." (P13)

## Discussion

This study explored various aspects of improving childbirth preparedness. Maternal health during pregnancy, having an antenatal care plan, improving health literacy, and developing a birth plan were identified as crucial factors for childbirth preparation. Facilitating factors included mental and emotional preparation, support, financial planning, participation in preparation classes, and awareness of the advantages and disadvantages of different childbirth methods. Hindrances included insufficient mental and emotional preparedness, inadequate support, weak antenatal care, information deficiencies, insufficient physical activity, and a lack of a birth plan. This study offers valuable insights for healthcare providers and policymakers to improve maternal and neonatal outcomes.

As mentioned, the findings of the study identified having an antenatal care plan and improving health literacy as crucial factors for enhancing childbirth preparations. A similar qualitative study by Kalisa et al. (2018) in rural Rwanda also recognized the role of receiving antenatal care as a facilitator of BP/CR from the viewpoints of healthcare workers and community members. Community health workers emphasized the importance of sending BP/CR messages through SMS alerts or during social gatherings to improve health literacy. Additionally, family support and health insurance were identified as other facilitators of BP/CR. On the contrary, disrespect and mistreatment of women by healthcare workers during childbirth, along with contradictory health policies such as charging fees for antenatal care and family planning services, as well as imposing fines on women who give birth outside health facilities, posed significant barriers to BP/CR [28]. The study by Kalisa et al. (2018) aligns with the present study regarding antenatal care programs, family support, and the importance of improving health literacy. It is important to note that the present study was conducted in health centers in Tabriz, Iran, where antenatal care is provided free of charge, and the majority of participants had health insurance coverage. As a result, the study did not mention the costs associated with receiving antenatal care or the need for health insurance. Furthermore, since all interviews took place in healthcare centers and hospitals at the participants' request, it is possible that the participants perceived the interviewer as part of the healthcare team, potentially influencing their reluctance to express views on healthcare worker misconduct or disrespect. WHO emphasizes the importance of respectful maternity care (RMC) to ensure a positive childbirth experience for women. Respectful maternity care is a fundamental human right and is essential for improving maternal and newborn health outcomes. The WHO’s recommendations for respectful maternity care include effective communication; respect and dignity; emotional support; informed consent; autonomy; dignity and accountability; and community involvement. By implementing these recommendations, the WHO aims to create a supportive and respectful environment for women during childbirth, ultimately improving maternal and newborn health outcomes [29, 30].

The importance of having an antenatal care plan and birth plan and improving health literacy, particularly raising awareness about danger signs during pregnancy, childbirth, and postpartum, as well as taking necessary action when such signs are observed, were highlighted in another qualitative study that examined the perceptions, experiences, and challenges of a rural Tanzanian community regarding BP/CR. The study's findings indicated that participants recognized the need to prepare for childbirth, attend antenatal clinics, seek hospital treatment for pregnancy and childbirth complications, and rely on family support for practical and financial preparations. They also mentioned relocating closer to hospitals and utilizing traditional herbs to achieve positive outcomes. Negative community attitudes towards unmarried pregnant women and transportation difficulties were identified as specific barriers to childbirth preparation, while poverty was recognized as a challenge. From the participants' perspective, although maternal healthcare is provided free of charge, the presence of informal costs associated with care creates problems for users [31]. The findings of this study are also aligned with the present study in many dimensions, including the antenatal care plan, taking necessary actions in case of pregnancy and childbirth complications, birth plan (financial planning and planning for hospital visits), and family member support. Due to the Islamic nature of Iranian society, unmarried pregnant women are either non-existent or very rare in the community, and there were no such individuals among the participants in this study. Therefore, negative societal attitudes towards unmarried pregnant women were not mentioned as a barrier to childbirth preparation in the present study.

The significance of improving health literacy and utilizing various information sources, including personal and others' experiences, healthcare providers, written resources such as books and the internet, virtual spaces, and social networks, was emphasized by most participants. The findings of a qualitative study on the utilization of social networks by partners of pregnant women for childbirth and parenthood preparation revealed that both close relationships (such as spouses, friends, and family) and less close relationships (such as other future parents) play a fundamental role as a source of social support during the process of childbirth and parenthood preparation. Partners are able to connect with others, share information, obtain reliable information, and prepare for childbirth and parenthood alongside the pregnant woman [32]. Additionally, a randomized controlled trial (RCT) of the pretest-post-test design examined the effect of a specific Android application on the knowledge of pregnant women's husbands about BP/CR. In this study, both the intervention and control groups received antenatal counseling, but only husbands in the intervention group installed the Android application. The application provided information to husbands during pregnancy about gestational age calculation, common complaints during pregnancy, danger signs, fetal growth and development, and pregnancy exercises. During childbirth, it provided information about danger signs, and in the postpartum period, it covered key danger signs in newborns and new mothers, infant care, breastfeeding techniques, and postpartum exercises. Reminders for antenatal and postpartum care were also included. The results indicated that BP/CR scores increased among couples after the intervention, both for participants in the intervention group and the control group. However, husbands who received the application showed a greater mean difference in BP/CR scores compared to those who received only counselling [33]. The findings of these studies align with the present study and support the use of various information resources and the support of husband, family members and others for childbirth preparation.

In the present study, the importance of husband involvement in care during pregnancy, childbirth, and the postpartum period was emphasized by most participants. They highlighted the role of husbands in providing emotional support, actively participating in decision-making, and assisting with birth planning. Consequently, husband support was identified as a facilitator of childbirth preparation. The findings of a qualitative grounded theory study on the involvement of pregnant women's male partners in BP/CR and the factors that enable or hinder their participation indicated that men play a significant role in supporting pregnant women and BP/CR. They assist in maintaining the health of pregnant women, provide financial support, participate in the decision-making process during treatment, arrange transportation, and facilitate the admission process to the hospital for pregnant women [34]. Similarly, the findings of another qualitative study conducted in the town of Arba Minch, located in southern Ethiopia, revealed that husbands actively participated in BP/CR program by fulfilling various roles. These roles included identifying the appropriate place for delivery, coordinating with a skilled birth attendant, recognizing signs of labor, obtaining accurate information about the due date, providing essential items such as food and clean clothes for both the baby and mother, acting as a personal companion, being knowledgeable about emergency conditions, responding promptly, saving money for emergencies, and identifying decision-makers in case of emergencies [35]. The findings of these studies align with the results of the present study, emphasizing the importance of having a birth plan, promoting health literacy, and involving husbands in the care process.

From the perspective of pregnant and postpartum mothers, factors such as lack of mental and emotional preparedness, lack of information, stress and fear regarding the natural childbirth process and its potential complications, limited support resources, inadequate antenatal care, and lack of a birth plan were identified as barriers of childbirth preparation. The findings of a systematic review (SR) on the factors influencing the implementation of BP/CR interventions revealed that adherence to traditional and incorrect beliefs by women and their families, scarcity of resources (including human resources), financial constraints for women and families, and a mismatch between the provided maternity care services and the desired services of mothers are significant barriers to childbirth preparation. The researchers of this study proposed that simultaneous access to skilled birth attendants across all levels of society and increased formal education for women could serve as facilitators of childbirth preparation [15]. The findings of this systematic review (SR) align with the perspectives of the mothers who participated in this study and highlight the importance of antenatal care and health literacy in improving childbirth preparation. Additionally, the study revealed the presence of weaknesses in care programs, financial challenges, and incorrect beliefs as barriers to effective childbirth preparation. The finding of a qualitative study on childbirth preparation from the perspectives of 36-40 week primiparous women, labor companions, and care providers in Lilongwe, Malawi indicated that primiparous women's lack of childbirth preparation stemmed from fear of the childbirth experience and uncertainty about pregnancy outcomes, ineffective traditional counseling, and inadequate antenatal education. Primiparous women exposed to traditional childbirth guidance rather than skilled care providers experienced childbirth fear and lacked adequate psychosocial childbirth preparation. Additionally, the study findings revealed that while labor companions may exacerbate childbirth stress, they also serve as crucial psychosocial support resources for primiparous women [36]. The findings of the aforementioned study align with the present study in identifying pregnant women's fear of childbirth and their information deficit as barriers to childbirth preparation and recommending the use of the mother's preferred labor companion.

The majority of the themes extracted from the present study align with and support the findings of the aforementioned studies. Exceptions include pregnancy health considerations and participation in childbirth preparation classes, which have not been mentioned in similar studies. Given that most studies examining childbirth preparation have been conducted in low- and middle-income countries, their findings emphasize the basic requirements for childbirth preparation, including improving family education and enhancing access to affordable antenatal care and skilled birth attendants. However, the present study was conducted in Iran, where the basic requirements for childbirth preparation are available to pregnant women, and the prevailing culture also places a strong emphasis on women's health during pregnancy, childbirth, and the postpartum period. For these reasons, the mothers participating in this study emphasized health considerations during pregnancy to improve childbirth preparation. In line with this, various studies have confirmed the impact of improved maternal nutrition [37], increased physical activity [38, 39], genital hygiene practices [40], oral hygiene [41, 42], and emotional well-being [43] on improving pregnancy and childbirth outcomes. Additionally, mothers mentioned participation in childbirth preparation classes, which have been offered by the Iranian Ministry of Health, and Medical Education since 2008, as a facilitator of childbirth preparation. Studies have shown that pregnant women's participation in these courses leads to improved lifestyle during pregnancy, childbirth, and the postpartum period [44]; increased positive experiences during pregnancy [45]; reduced stress and anxiety [46]; decreased fear of childbirth [47]; achieving a positive mindset towards labor pain; better involvement in the childbirth process; improved decision-making by correcting misconceptions; increased self-confidence; awareness of childbirth options; and reduced need for pain relief during childbirth [48].

## Strengths and limitations of the study

Based on the conducted searches, this study is the first qualitative study in Iran to examine the perspectives of pregnant and postpartum women on childbirth preparation and its facilitating and inhibiting factors. To obtain comprehensive views on childbirth preparation, attempts were made to include a diverse range of participants.

One limitation of this study was social desirability bias, meaning that participants might have provided socially desirable responses rather than genuine ones. They may have refrained from expressing negative opinions about the behavior of healthcare providers due to perceiving the interviewer as part of the healthcare system. To address this issue, the interviewer assured participants that she was not part of the healthcare team and that their identities would be kept confidential, especially when discussing sensitive topics. This encouraged them to express their true perspectives. Another limitation of this study was the presence of the infant in the mother's arms during interviews with postpartum women, which sometimes led to interruptions and disruptions during the interviews.

## Application of study findings

By integrating the findings of this study regarding the perspectives of pregnant and postpartum women on childbirth preparation and its facilitating and inhibiting factors with the results of a review of relevant literature and studies in this field, along with considering the perspectives of maternal health specialists, supportive strategies can be developed to enhance childbirth preparation in accordance with the regional culture and made available to healthcare policymakers. Among these support strategies, we can mention the following items: increasing the availability and accessibility of childbirth preparation classes, including online options for those who cannot attend in person; organizing family-oriented childbirth education sessions to involve spouses and other family members; providing access to counseling services to address fears and emotional concerns related to childbirth; promoting open communication between the expectant mother and her support network to ensure emotional and practical support; encouraging discussions with healthcare providers to help expectant mothers make informed decisions about their childbirth options; and providing information on available financial assistance programs and insurance coverage for maternity care. Providing these supportive strategies in the form of guidelines to key stakeholders, including healthcare providers, will lead to their implementation in antenatal counseling and antenatal care programs to improve childbirth preparation among mothers, which aligns with the goal of the population rejuvenation policy adopted in Iran.

## Conclusion

In this study, pregnant and postpartum women highlighted several aspects to improve childbirth preparation, including: paying attention to health-related issues during pregnancy; following an antenatal care plan; increasing health literacy; and planning for childbirth. From the mothers' perspectives, facilitators of childbirth preparation included psycho-emotional preparedness, support from spouses and other family members, financial planning, participation in childbirth preparation classes, and awareness of the benefits and challenges of different types of childbirth. Conversely, barriers to childbirth preparation included a lack of psycho-emotional preparedness, inadequate support resources, a weak antenatal care plan, insufficient information, obstacles to exercise and physical activity, hindrances to participation in childbirth preparation classes, fear of natural childbirth, the belief in the inability to have a natural childbirth, and the absence of a birth plan. Based on the findings of the current study, it is suggested to conduct more research on investigating childbirth readiness from the perspectives of mothers’ partners and healthcare providers; examining the effectiveness of various childbirth education programs in enhancing women’s readiness for childbirth; studying the role of healthcare providers in preparing women for childbirth; and examining the role of technology, such as mobile apps and online resources, in enhancing childbirth readiness.

### Supplementary Information

Supplementary Material 1.

## Data Availability

All relevant data are given within the manuscript.

## Abbreviations

FoC: Fear of childbirth
PTSD: Post-traumatic stress disorder
CD: Cesarean delivery
WHO: World Health Organization
BP/CR: Birth preparedness and complication readiness
SDG3: Sustainable Development Goal 3
CRS: Childbirth readiness score
RMC: Respectful maternity care
